# A graph-theoretic framework for integrating mobility data into mathematical epidemic models

**DOI:** 10.1016/j.idm.2025.02.008

**Published:** 2025-02-15

**Authors:** Razvan G. Romanescu

**Affiliations:** a Department of Community Health Sciences, University of Manitoba, Canada; b Center for Healthcare Innovation, University of Manitoba, Canada

**Keywords:** Infectious disease models, SIRS, Epidemics on networks, Cell phone mobility, Non-homogeneous mixing

## Abstract

Advances in modeling the spread of infectious diseases have allowed modellers to relax the homogeneous mixing assumption of traditional compartmental models. The recently introduced synthetic network model, which is an SIRS type model based on a non-linear transmission rate, effectively decouples the underlying population network structure from the epidemiological parameters of disease, and has been shown to produce superior fits to multi-wave epidemics. However, inference from case counts alone is generally problematic due to the partial unidentifiability between probability of person to person transmission and the average number of contacts per individual. An alternate source of data that can inform the network alone has the potential to improve overall modeling results. Aggregate cell phone mobility data, which record daily numbers of visits to points of interest, provide a proxy for the number of contacts that people establish during their visits. In this paper, we link the contact rate from an epidemic model to the total number of contacts formed in the population. Inferring the latter from Google Community Mobility Reports data, we develop an integrated epidemic model whose transmission adapts to population mobility. This model is illustrated on the first four waves of the COVID-19 pandemic.

## 1 Introduction

Mathematical infectious disease models typically produce one curve that describes the trajectory of an epidemic, based on a set of parameters. The model is fit to observed cases of infection, producing a best fitting curve, which is assumed to describe the overall epidemic progression in the past and (near) future. In reality, parameters governing transmission change, as individuals adapt to real or perceived risks of the circulating pathogen. Public health agencies introduce restrictions on mobility and access, aiming precisely to change relevant transmission parameters and contain the infection. As a result, datasets of observed infection counts are a realization of models changing in real time. One solution is to fit piecewise models according to known restrictions. This model building approach amounts to adding parameters and relying on the data to infer the best fitting values for different time segments of the epidemic (Romanescu et al., 2023; Wu et al., 2021). The shortcoming of this approach is that it relies on a single source of data, i.e., case counts, to infer changes in both the epidemic, as well as the social network processes. This can lead to confounding and identifiability problems, as changes in either process can lead to the same predicted case counts. For example, both a less infectious strain of the pathogen, or fewer social contacts will result in fewer observed cases of disease. It would be very desirable to have different types of data in addition to case counts, which can be used to identify one of these processes in isolation.

Recently, cell phone mobility data has emerged as a promising source of information to infer the contact network underlying disease transmission. The utility and far-reaching implications of using cell data to study human mobility on a large scale has been noted a decade ago in Becker et al. (2013), who used this data to describe the daily commute and travel patterns of the metropolitan populations of Los Angeles, San Francisco, and New York. A review of the different types of data that can be generated from mobile phones is given in Grantz et al. (2020). It becomes necessary to distinguish between granular versus aggregate mobility data sources. The focus of this paper will be the latter, although it is worth mentioning how highly granular data has been used in epidemic modeling. During the COVID-19 pandemic, spatial cell phone locations have been used to inform movement of individuals between points of interest and census block groups, in a bipartite graph with weighted edges (Chang et al., 2021). In general, dealing with the vast amounts of data available from granular sources is challenging and requires some form of aggregation or dimension reduction before this data becomes interpretable in a modeling setting. For instance, Levin et al. (2021) uses machine learning techniques to reduce mobility data to a small number of dimensions, which are then used to identify mobility patterns, subpopulations and correlations with COVID-19 cases.

Once the data has been compacted into a (multivariate) time series format, either by oneself or a data provider, the problem of integration into classical compartmental models of infectious disease becomes feasible. Google Community Mobility Reports (GMR) provide a single time series of changes in aggregate mobility volume for one location type (Aktay et al., 2020). There have been a few attempts in the literature so far to assimilate this data, often using mobility to explain changes in the efective reproduction number ($R_{t}$). For instance, Li et al. (2021) have used the different component time series of the GMR in a multivariate log-linear regression to predict changes in $R_{t}$ at UK local authorities. Sulyok and Walker (2020) have used the GMR data within generalised additive models to explain case incidence. A more mechanistic approach was taken by Vanni et al. (2021) who derive $R_{t}$ based on a chemical representation of infectious diseases, as two types of molecules colliding in a solution; importantly, the contact rate increases linearly with movement speed of individuals, and this is informed by GMR. The approach we take in this paper similarly models $R_{t}$ in terms of the GMR data, with the crucial difference that we link both the transmission rate in the epidemic model, as well as the total volume of connections given by GMR, to the underlying network architecture of individuals. The mechanism is that reductions in mobility make the network more sparse, which affects the transmission rate in a (possibly) nonlinear fashion. A related problem to data integration is how mathematical models handle changes in transmission rates, with the most common approach being a proportional shift (see e.g., Anderson et al., 2020). Our approach is flexible in this regard, and we can accommodate common and custom changes to the transmission rate, which can be used to evaluate different reduction policies.

In the rest of the paper, we first review an SIRS epidemiological model based on a non-linear transmission function, in Section 2. We then derive the graph-theoretic link between the transmission function, which can also be interpreted as the average contacts of the infectious set, and mobility data, which acts as a proxy for the total number of edges in the population network. The two sources of data – namely case counts and aggregate mobility, – then inform an augmented epidemic model whose transmission rate changes in response to mobility volumes, depending on which individuals are impacted more by mobility restrictions. We fit this model to COVID-19 data from New York in Section 3 to illustrate how including GMR data helps inference. We conclude with a discussion and recommendations for future research and data collection in Section 4.

## 2 Methods

### 2.1 Epidemiological model

The epidemic model we use follows the synthetic network model, a discretized ODE-type model of transmission in the susceptible-infected-removed-susceptible (SIRS) setting. This model has been introduced elsewhere (Romanescu et al., 2023), and is summarized in this section. The distinguishing feature of this framework is that it models daily incidence ($y_{t}$), as opposed to prevalence ($I_{t}$), via the effective reproduction number ($R_{t}$). Let $w_{s}$ be the probability mass function of the time (in days) between a primary and secondary infection, i.e., the generation interval. Then we can estimate incidence at time t+1 as (Abbott et al., 2020; Nishiura & Chowell, 2009)

(1) $$y_{t+1}=R_{t}\sum_{s=1}^{M}w_{s}y_{t+1-s}\text{,}$$

where M is the maximum range of the generation interval. $R_{t}$ can be decomposed, as per standard theory, into $R_{t}=\alpha \times S_{t}\times c_{t}$, where α is the person to person probability of transmission given that a contact exists; $S_{t}$ is the fraction of all nodes that are still susceptible; and $c_{t}$ is the average number of contacts of infectious individuals at time t, less one (the previous infector is excluded). The model additionally assumes that $c_{t}=c\left(S_{t}\right)$ where c(∙) is a decreasing function in the SIR case, meaning that the average contact rate of currently active infectors is a one-to-one function of $S_{t}$. This relationship emerges in a number of population structures, for instance in well-mixed networks (as discussed in the next subsection); or in non-network models of randomly-mixing individuals with heterogeneous susceptibility (Novozhilov, 2008). The assumption of a well-mixed population (not to be confused with a homogeneous population) is important, so this model may not work well in a structured population, such as loosely-connected clusters (as may be the case in a rural setting, for instance). Thus, $R_{t}$ is computed as:

(2) $$R_{t}=\alpha \times S_{t}\times c\left(S_{t}\right)\text{.}$$

This is used to sequentially compute $y_{t+1}$ from Equation (1), then update the susceptible fraction as

(3) $$S_{t+1}=S_{t}-\frac{y_{t+1}}{\rho_{t}N}\text{,}$$

where N is the total population size and $\rho_{t}$ is the underreporting rate, defined as the fraction of reported cases to actual infections.

An essential advantage of factorization (2) is that it not only separates the effect of the pathogen (α) from the effect of the transmission network, but also decouples potential infectious contacts into an epidemic state variable ($S_{t}$), and a component that only reflects network architecture (function c(∙)). This will be important for the rest of this paper because we can model changes in the social network due to factors exogenous to disease spread, via shifts in curve c(∙).

Among the choices for contact rate function given in Romanescu et al. (2023), we select $c\left(S_{t}\right)=\alpha^{-1}a_{1}e^{-a_{2}{\left(1-S_{t}\right)}^{a_{3}}}$, a model adapted from Granich et al. (2009), where $a_{1}$, $a_{2}$, and $a_{3}$ are parameters estimable from data. This was shown in our previous work to outperform the mass action model (in which $c\left(S_{t}\right)=\text{constant}$) by a wide margin in terms of quality of fit to data. Note that α and c(∙) are only identifiable as a product and not individually, and we will denote the estimable function by $\tilde{c}\left(S_{t}\right)=\alpha c\left(S_{t}\right)$. For clarity of exposition, we will be using the tilde notation throughout the paper to indicate the estimable version of a variable or function.

In the SIRS case, the dynamics of transmission can be approximated (Romanescu et al., 2023) via an adjusted c(∙). Assume that a fraction ν of infected individuals lose their immunity to the pathogen at the beginning of each new wave. Let $S_{0}$ and $S_{-1}$ represent the overall fraction $S_{t}$ at the beginning of the current epidemic wave, and at the end of the previous wave, respectively, such that $S_{0}=S_{-1}+\nu \left(1-S_{-1}\right)$. We use the following weighted contact rate in each subsequent wave of the epidemic:

(4) $$c^{w}\left(\frac{S_{t}}{S_{0}}\right)=w_{A}c\left(S_{-1}\times \frac{S_{t}}{S_{0}}\right)+w_{B}c\left(\frac{S_{t}}{S_{0}}\right)\text{,}$$

where the weights $w_{A}=\frac{S_{-1}\left(1-\nu \right)}{S_{0}}$ and $w_{B}=\frac{\nu }{S_{0}}$ sum to 1. Note that $S_{0}$ and $S_{-1}$ need to be reset at the beginning of each new wave. The effective reproductive number is then computed as $R_{t}=\alpha S_{t}c^{w}\left(S_{t}/S_{0}\right)$, and $S_{t}$ is updated in the same way as in the SIR case. Using a weighted contact rate is motivated by the fact that newly susceptible individuals (moving from R → S) tend to have more contacts compared to those left susceptible at the end of the previous wave, as they have gotten infected before. The choice of weights reflects the fact that the overall degree distribution of susceptibles at the start of a new wave is a mixture of distributions between the degree of those left at the end of the previous wave, and the original degree distribution (in the fully susceptible population), with weights equal to $w_{A}$ and $w_{B}$, respectively.

Finally, each wave beyond the first is allowed a different person-to-person transmissibility via the relative change parameters $v_{2},v_{3},\ldots$, to reflect changing infectiousness in the pathogen strains. Thus, transmissibilities in each wave are $\alpha ,\alpha v_{2},\alpha v_{3}\text{,}$ etc.

### 2.2 Modeling the reduction in social connectivity as a result of public health orders

#### 2.2.1 Link between total contacts in the population and the contact rate function c(t)

In the epidemic model described above, it is possible to recover the total number of contacts in the population from the contact rate function of the infectious set. Assume a disease with α=1 that progresses in discrete, non-overlapping generations in the SIR framework, and that all infectious individuals recover at the end of their generation. Formally, let the population be represented by a fixed, connected graph G=(X,E) with edge set E and vertex set X, with cardinalities N=|X| and E=|E|. Let $J_{t}$ be the set of infectious vertices (individuals) at time (or generation) t, and $E_{t}$ be the set of all edges touching individuals in $J_{t}$. We denote the susceptible set at time t to be $S_{t}=X\setminus \overset{t}{\underset{u=1}{⋃}}J_{u}$. The numbers of elements in the random sets $J_{t},E_{t}$, and $S_{t}$ are random variables $I_{t},E_{t}$, and $S_{t}$, respectively. According to the definition in the previous subsection, $c_{t}=\frac{E_{t}}{I_{t}}-1$, assuming that individuals in $J_{t}$ are not themselves sharing common edges. We further assume, as above, that there exists a function c(∙) such that $E\left[c_{t}|S_{t}\right]=c\left(S_{t}/N\right)$. In other words, that $c_{t}$ depends only on cumulative infections up to time t, and not on the pathways of infection; formally, that $c_{t}|S_{t}$ is independent of $J_{0},J_{1},\ldots ,J_{t}$. Such a function c(∙) has been shown to describe the mean-field behavior of diffusion processes over first order networks, i.e. networks characterized by degree distribution alone, with no higher order structure (Newman, 2002; Romanescu & Deardon, 2017; Volz, 2008). The relationship also seems to hold empirically in networks with second order structure, or joint distribution of degrees of connected individuals, as illustrated in simulation studies (Romanescu, 2024), though this has not been formally proved yet.

Thus, we have, by definition, that $\left(c_{t}+1\right)I_{t}=E_{t}$, for t=0,…,T, where T is the last time that an infection is recorded. Adding all these equations side by side, we get

(5) $$\sum_{t=0}^{T}E_{t}=\sum_{t=0}^{T}\left(c_{t}+1\right)I_{t}\approx \sum_{t=0}^{T}\left[c\left(\frac{S_{t}}{N}\right)+1\right]\left(S_{t-1}-S_{t}\right)=N\;\sum_{t=0}^{T}\left[c\left(s_{t}\right)+1\right]\left(s_{t-1}-s_{t}\right)\approx N\int_{s_{T}}^{1}\;c\left(s\right)+1ds\text{,}$$

where we used the notation $s_{t}=S_{t}/N$ (in the rest of the paper except for this section, $S_{t}$ will continue to denote the fraction of susceptibles, as their full number will not be needed again).

As this is a connected graph, a probability of per-edge infection α=1 necessarily implies that all individuals in the population will become infected by time T. Thus, we have $S_{T}=0$, $\overset{T}{\underset{t=1}{⋃}}J_{t}=X$, and also $\overset{T}{\underset{t=1}{⋃}}E_{t}=E$. A few interesting observations can be made about equation (5). On the right hand side, we get a constant, which depends only on the shape of function c on the interval [0,1]. For the sum on the left-hand side, notice that every edge in the graph is counted in exactly two consecutive $E_{t}$ sets, as per our setup: once when the first end of the edge gets infected, and once at the next time point, when infection passes through the edge to the other end. Thus, the left-hand side is also constant, as $\sum_{t=0}^{T}E_{t}=2E$. Finally, notice that neither end of Equation (5) depends on the choice of initial infections set $J_{0}$, although the terms in the middle (such as $I_{t}$) are random variables.

It will be more convenient for the future to speak in terms of the average degree (D) of the graph instead of the total number of edges. This represents the mean number of contacts (degree) of a random individual in the population, and obeys the identity $E\left[D\right]=\frac{2E}{N}$; thus, we can write $\sum_{t=0}^{T}E_{t}=NE\left[D\right]$. Substituting in (5), we conclude that

(6) $$E\left[D\right]=\int_{0}^{1}\;c\left(s\right)+1ds\text{.}$$

Notice that this formula is a feature of the network, and does not depend on the values of the epidemiologic parameters. Equation (6) is important because we can estimate E[D] from non-epidemic sources (such as mobility data), and infer how the contact rate changes as a direct result of observed changes in the transmission network. While traditional epidemic models relying on infection data alone cannot easily separate c(∙) from the transmissibility α, which confounds it, equation (6) provides a way to tease out the magnitude of c(∙) independently of α.

#### 2.2.2 Modeling changes over time in the shape of the contact rate function

**Fig. 1:**
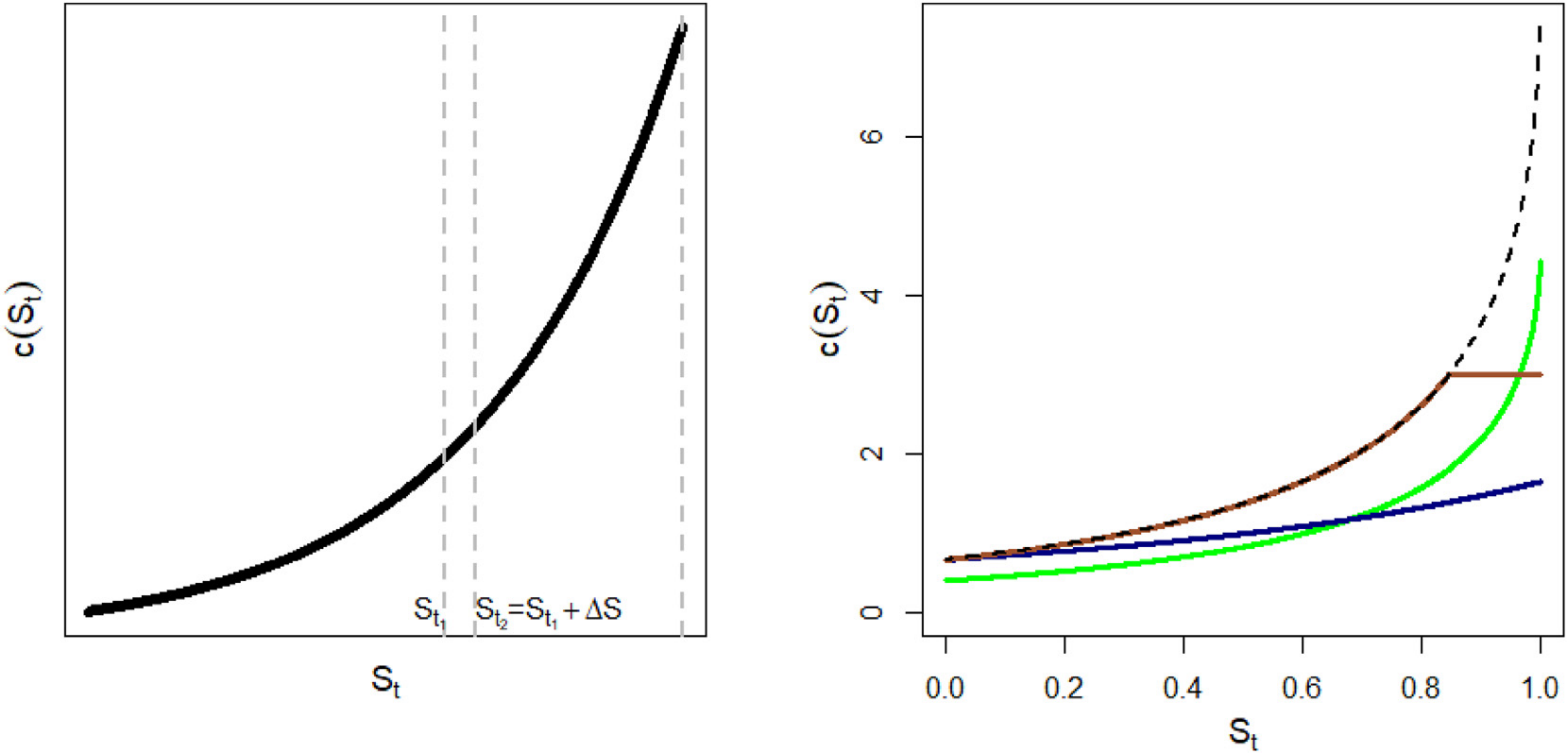
(Left) Contact rate of the infectious set during an epidemic, as a function of the remaining susceptible fraction. The area of a slice under curve c(∙) between $S_{t_{1}}$ and $S_{t_{2}}$ gives the average number of contacts (less 1) of typical infected individuals between epidemic ages $S_{t_{1}}$ and $S_{t_{2}}\text{.}$ (Right) Changes in the original curve c(∙) (dashed line) according to the scenario: downscaled (green), tapered (blue), top off (sepia).

As our investigation focuses on changes in contact volumes over time, we need to model ways in which the contact rate c(∙) changes on a daily basis. Firstly, quantities D and c(∙) should be viewed as time-dependent, because they change in real-time as the network changes (we use $D_{t}$, and, by abuse of notation, $c_{t}\left(∙\right)$ to refer to the current state of the non-epidemiologic network on day t). Secondly, equation (6) relates $D_{t}$ to the area under $c_{t}\left(s\right)$, at each point in time; but we also need to postulate how the shape of $c_{t}\left(∙\right)$ is allowed to change with t. Indeed, reducing the contact rate is a crucial objective of public health policy and comes in the form of social restrictions, such as lockdowns and capacity limits. Being able to correctly model $c_{t}$ is thus paramount for evaluating the effectiveness of control measures. As discussed in the introduction, there are a few approaches in the literature on how $c_{t}$, or the transmission rate, might change, although by far the most common is via multiplication by a factor. This approach is used in modeling, often by default, because epidemic data alone does not usually permit one to distinguish between a drop in transmissibility of the pathogen, and a restriction on individual contacts; thus, there is rarely a basis for a more complex form. However, working specifically with network models, we have shown that a “tapered” approach might make more sense when there is heterogeneity in contacts (Romanescu et al., 2023). This consists of adding an exponential tail to the probability mass function of individual contacts. More exactly, if we let $f_{1}$ and $f_{2}$ denote the fractions of individuals in the original population with $i_{1}$ and $i_{2}$ contacts ($i_{1}<i_{2}$), then, under restrictions, the new fractions $f_{1}^{*}$ and $f_{2}^{*}$ satisfy $\frac{f_{2}^{*}}{f_{1}^{*}}=\varphi^{i_{2}-i_{1}}\frac{f_{2}}{f_{1}}$, where φ<1 controls the severity of restrictions on the network. This assumption can be shown to imply the tapered scenario in Table 1, under a first order network model. This scenario can be argued to be more realistic than a scale reduction, at least in certain situations. Finally, scenario 3 consists of capping average connections at a certain level. While this is not necessarily a realistic representation of past containment efforts, we would expect it to be the most effective among the three scenarios, if it could be implemented. In each scenario in Table 1, the time-dependent variable $K_{t}$ quantifies the strength of restriction on the original (unrestricted) network. Fig. 1 graphically illustrates the shift in c(∙) for each scenario. While this is by no means an exhaustive list, it does capture a range of possibilities, each having different implications for the total burden of disease, as will be illustrated in the data application.

**Table 1:** Scenarios of reduction in contact rate function.

Scenario	Change relative to unconstrained c(∙)	Individual level impact
1. Scaled down	$c_{t}\left(S_{t}\right)=K_{t}c\left(S_{t}\right)$	Individuals “equally” impacted, i.e., everyone loses the same percentage of connections
2. Tapered	$c_{t}\left(S_{t}\right)=c\left(K_{t}S_{t}\right)$	Highly connected individuals disproportionately restricted compared to least connected
3. Top off	$c_{t}\left(S_{t}\right)=c\left(\min\left(S_{t},K_{t}\right)\right)$	Individuals affected tend to have connections above a certain level.

### 2.3 Pre-processing Google Community Mobility Reports data

The Google Community Mobility Reports (GMR) were published between February 2020 and October 2022 and report the change in cell phone traffic volume at different categories of locations, including: work, retail and recreation, parks, public transit, and residential. Changes are reported as percentages compared to a reference baseline defined as the median volume for that day of the week, over a period of 5 weeks between Jan 3–Feb 6, 2020 (Google LLC). Numbers are aggregated to preserve confidentiality, and no absolute values are given.

We pre-process the Community Mobility Reports to infer the average number of hours spent in various location categories, accounting for differences between weekdays and weekends. From the American Time Use Survey (ATUS) we estimate the daily time spent in each mobility category (Table A1 in the Appendix). A similar approach was taken in Gutiérrez-Jara et al. (2022), who produce detailed simulations to show how changes to daily patterns of activity might affect epidemic spread. Since the GMR data are given as percentage changes relative to the same day of the week during Feb 2020–March 2020, we multiply the relative fraction by the number of hours spent in each category from ATUS to obtain the estimated actual hours spent in the activity. We do this separately for weekends and weekdays. In the end, for each category apart from residences and parks, i.e., for retail & recreation, grocery & pharmacy, transit stations, and workplaces, we now have a time series of hours spent at that location on each day, which we jointly denote as vector $H_{t}=\left(H_{t}^{E},H_{t}^{G},H_{t}^{T},H_{t}^{W}\right)$, for each day t=1,…,T.

### 2.4 Joint inference from epidemic and mobility data

#### 2.4.1 General setup in the SIR case

In what follows, we will let the Mobility Reports inform restriction level $K_{t}$ on each day. To calibrate $K_{t}$ we equate the number of total contacts lost (as the difference in area under the curves c and $c_{t}$) with the reduction in cell phone traffic volume.

Assume that individuals establish epidemiologically relevant contacts at rates $\beta^{E},\beta^{G},\beta^{T},\beta^{W}$ per hour spent in each category, and constant rate $\beta^{H}$ contacts at home. Thus, if contacts were established uniformly and individuals were homogeneous, the average number of contacts formed on day t is ${\beta^{E}H}_{t}^{E}+\beta^{G}H_{t}^{G}+\beta^{T}H_{t}^{T}+\beta^{W}H_{t}^{W}+\beta^{H}$, where we assume that contacts at home are independent of time spent. A similar regression approach was taken in García-Cremades et al. (2021), who have also pointed out the non-linearity and multicollinearity present in the data, which violate the assumption of the regression model. To alleviate the multicollinearity issue, we de-trend each $H_{t}$ variable by first computing the trend $h_{t}^{trend}$ as the 7 day moving average of the sum of $H_{t}$ ‘s in the four categories noted above, then subtracting a scaled trend from each variable as

$$h_{t}^{A}=H_{t}^{A}-\frac{\bar{H_{t}^{A}}}{\bar{h_{t}^{trend}}}h_{t}^{trend}\text{,}$$

for A∈{E,G,T,W}, such that each $h_{t}^{A}$ is de-trended and centered. We write the average number of contacts now as $h_{t}∙\beta +\beta^{H}$, where $h_{t}=\left(h_{t}^{trend},h_{t}^{E},h_{t}^{G},h_{t}^{T},h_{t}^{W}\right)$ and we allow an extra parameter for trend in $\beta =\left(\beta^{trend},\beta^{E},\beta^{G},\beta^{T},\beta^{W}\right)$.

Non-linearity in translating between cell phone traffic and number of infectious contacts formed should not be surprising. Just as the epidemiologic model is non-linear, it is possible, even likely, that equal, incremental changes in overall mobility volume may not result in equal increments of contacts gained. For example, we may postulate that a reduction in cell phone traffic from 100 to 95% is driven by individuals who willingly choose to reduce their mobility in response to a circulating pathogen (perhaps because they are more proactive, or risk-averse), whereas a reduction in volume from 55 to 50% is more likely driven by individuals who take a “wait and see” approach, or who were constrained by law. If the number of contacts formed correlates with behavioral pattern, then the relationship will be non-linear. To accommodate this, we define the mobility metric as $M_{t}=l_{lower}+\left(l_{upper}-l_{lower}\right)f\left(h_{t}∙\beta +\beta^{H}\right)$, where f is the standard logistic curve. During fitting, $\beta^{H}$ will act as a baseline level that determines where the points $h_{t}∙\beta$ fall on the logistic curve, i.e., whether the relationship is concave or convex; as such, $\beta^{H}$ will not be interpretable as number of contacts formed at home.

By identifying $E\left[D_{t}\right]=M_{t}$ in Equation (6), we can write:

$$\int_{0}^{1}c_{t}\left(s;\;K_{t}\right)ds+1=l_{lower}+\left(l_{upper}-l_{lower}\right)f\left(h_{t}∙\beta +\beta^{H}\right)\text{,}$$

or, using the estimable function $\tilde{c}\left(s\right)=\alpha c\left(s\right)$,

(7) $$\int_{0}^{1}{\tilde{c}}_{t}\left(s;\;K_{t}\right)ds={\tilde{l}}_{lower}+{\tilde{l}}_{upper}f\left(h_{t}∙\beta +\beta^{H}\right)≝{\tilde{M}}_{t}\text{,}$$

where ${\tilde{l}}_{lower}=\alpha \left(l_{lower}-1\right)$ and ${\tilde{l}}_{upper}=\alpha \left(l_{upper}-l_{lower}\right)$. Note how α blends in naturally with other unknowns, and will not require separate estimation. We further denoted the right hand side by ${\tilde{M}}_{t}$, which will be the estimable mobility metric, in the absence of information on α. Parameters ${\tilde{l}}_{lower}$ and ${\tilde{l}}_{upper}$ will be chosen as a function of curve $\tilde{c}$ parameters in order to ensure that Equation (7) is solvable for $K_{t}$ at each time point t, as detailed under ‘Model implementation details’ in the Appendix. The β parameters will be estimated as part of the model fit.

Fitting the full model proceeds in two steps. In the first, integral equation (7) is solved for $K_{t}$ as a function of the other model parameters and of vector $h_{t}$, for a specific reduction scenario in Table 1. The solution $K_{t}^{*}$ gives the amount of reduction in contact rate consistent with the observed mobility for day t. In the second step, all free model parameters are estimated via maximum likelihood, as detailed in the Appendix.

#### 2.4.2 Solution for scale down scenario

In this case, $c_{t}\left(S;\;K_{t}\right)=K_{t}c\left(S\right)$ and Equation (7) becomes $K_{t}\int_{0}^{1}\tilde{c}\left(s\right)ds={\tilde{M}}_{t}$. We first solve for $K_{t}$ as $K_{t}^{*}=\frac{{\tilde{M}}_{t}}{\int_{0}^{1}\tilde{c}\left(s\right)ds}$. Then, under restrictions, the epidemiologic model (2) becomes

(8) $$R_{t}=S_{t}\times K_{t}^{*}\times \tilde{c}\left(S_{t}\right)=S_{t}\times \frac{{\tilde{M}}_{t}}{\int_{0}^{1}\tilde{c}\left(s\right)ds}\times \tilde{c}\left(S_{t}\right)\text{.}$$

#### 2.4.3 Solution for tapered scenario

In this case we have $\int_{0}^{1}\tilde{c}\left(s;\;K_{t}\right)ds=\frac{\int_{0}^{K_{t}}\tilde{c}\left(s\right)ds}{K_{t}}$ (see Appendix for derivation). Equation (7) thus becomes

(9) $$\int_{0}^{K_{t}}\tilde{c}\left(s\right)\text{ds}=K_{t}{\tilde{M}}_{t}\text{.}$$

This requires a numerical solution for $K_{t}$, though the integral can be expressed via special functions for our choice of $\tilde{c}$, hence simplifying the calculation (see Appendix for details). Denoting $K_{t}^{*}$ the solution of (9), the restricted epidemiologic model becomes $R_{t}=S_{t}\times \tilde{c}\left(K_{t}^{*}S_{t}\right)$.

#### 2.4.4 Solution for top off scenario

Here, $K_{t}$ is the level of the susceptible fraction from which the contact rate tops off. Equation (7) leads to

(10) $$\int_{0}^{K_{t}}\tilde{c}\left(s\right)ds+\left(1-K_{t}\right)\tilde{c}\left(K_{t}\right)={\tilde{M}}_{t}\text{.}$$

Solving (10) numerically for $K_{t}^{*}$, the effective reproductive number becomes $R_{t}=S_{t}\times \tilde{c}\left(\min\;\left(K_{t}^{*},S_{t}\right)\right)$.

#### 2.4.5 Inference in the SIRS case

In the SIRS case, Equation (7) does not change, as it is independent of infection or reinfection status. So the solution $K_{t}^{*}$ for each time t is the same as in the SIR case. However, under reinfection, $\tilde{c}$ changes during subsequent waves according to formula (4). Thus, when computing $R_{t}$ as a function of $S_{t}$ and $K_{t}^{*}$, we will be using the weighted ${\tilde{c}}^{w}$ which accounts for reinfections. Note, in particular, in Equation (8), the middle term uses $\tilde{c}$ as in the SIR case (as this is part of $K_{t}^{*}$), but the SIRS version ${\tilde{c}}^{w}$ in the last term.

## 3 Results

We illustrate this framework on the time series of COVID-19 cases from New York State, over the period March 2020–February 2022, which is comprised of four visible waves. The dataset, including the exact definition of waves, is described elsewhere (Romanescu et al., 2023). This population was chosen because our previous research found evidence of heterogeneity in contacts, specifically a power law degree distribution (Romanescu & Deardon, 2017), which makes the contact rate very different from a flat curve. Being a highly urban area, this satisfies the assumption of a well-mixed population, which is required for this type of epidemic model. Other features that makes this dataset a good example include the fact that all four waves of COVID-19 are well-represented (compared to some other cities where the first wave is absent), and there is an extensive subway system, leading us to expect that the Traffic category in GMR will be more prominent in transmission, compared to cities with a less developed public transit infrastructure.

Data obtained from Google Community Mobility Reports for the corresponding period is preprocessed as described in Methods. Other epidemiological parameters (including the underreporting rate $\rho_{t}$) are taken from Romanescu et al. (2023) and references therein. We further consider an offset vector giving the delay, for each day, between GMR and COVID reported cases, which is computed as: ${delay}_{t}={incubation}_{t}+case\_delay-GMR\_delay$. Here, incubation periods are specific for each strain (Wu et al., 2022): for wildtype 6.65 days, for alpha variant 5.00 days, for delta 4.41 and for omicron 3.42 days. Reporting delay of COVID cases is estimated to be 3.3 days during the summer of 2020 (Harris, 2022), while mobility data is assumed to have a delay of 2 days (Aktay et al., 2020).

### 3.1 Model fit to the NY dataset

**Fig. 2:**
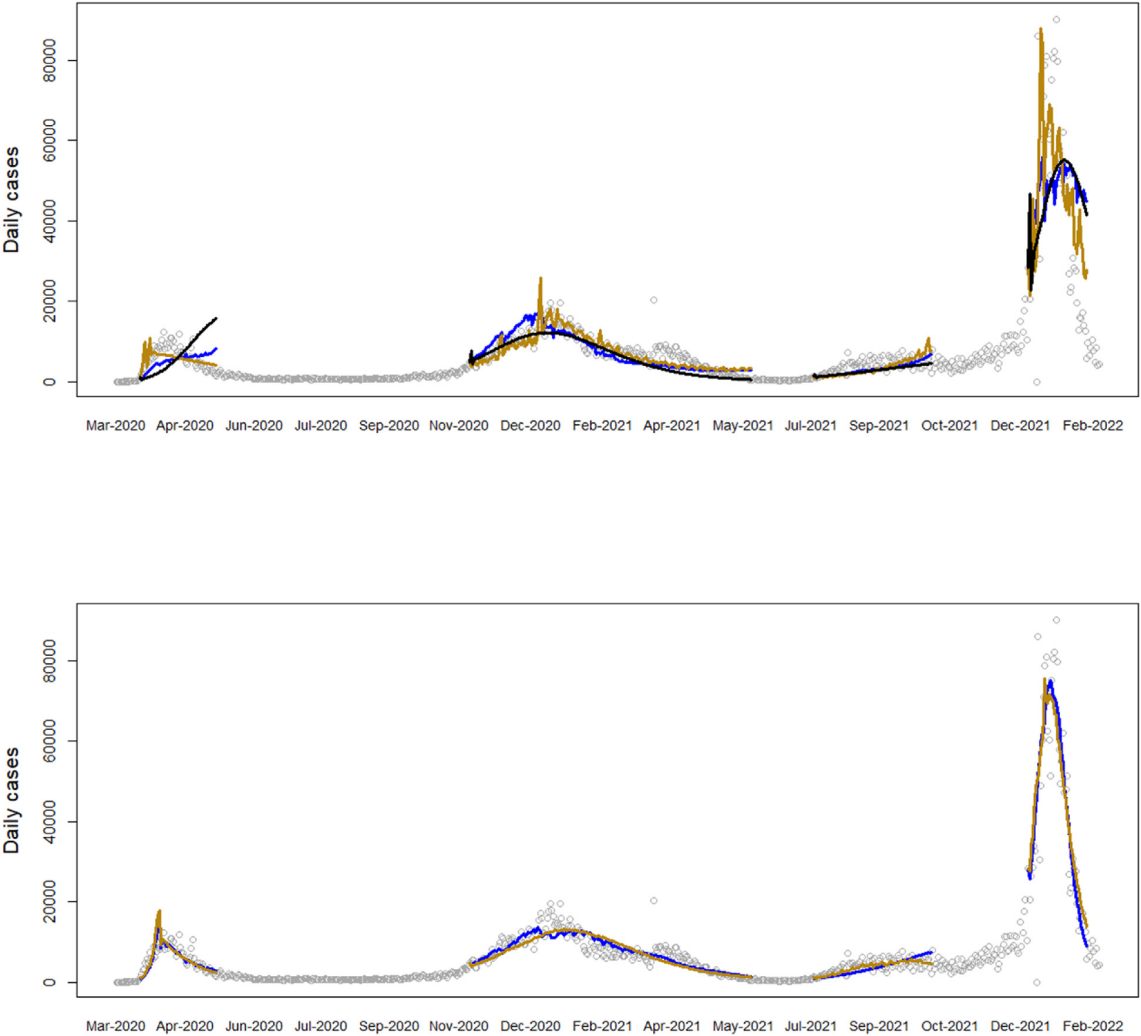
Fit of the curve model to the NY case data under different reduction scenarios: (upper) no reduction (black), downscaled (blue), tapered (gold). (Lower) downscaled (blue), and tapered scenarios (gold), allowing for an extra parameter ($\beta_{0}$) in ${\tilde{M}}_{t}$.

We first fit the assumed curve c(∙) without any restrictions to the entire dataset – this fit does not use the mobility data. We then fit the model including the GMR data, for each of the first two reduction scenarios in Table 1. Fig. 2 shows the fitted curves, and parameter estimates are given in Table 2.

**Table 2:** Parameter estimates of the fits and negative log likelihood values for each assumed reduction scenario.

Parameters		No reduction	Scaled down	Tapered	Scaled down (incl. $\beta_{0}$)	Tapered (incl. $\beta_{0}$)
$\tilde{c}\left(S_{t}\right)$	$a_{1}$	20 (−)	20 (−)	20 (−)	3.09 (0.35)	19.99 (0.65)
	$a_{2}$	3.21 (0.06)	2.38 (0.13)	3.04 (0.04)	300 (−)	3.58 (0.01)
	$a_{3}$	0.035 (0.004)	0.026 (0.006)	0.650 (0.191)	4.527 (0.111)	0.179 (0.005)
loss of immunity rate	ν	0.057 (0.018)	0.087 (0.030)	0 (−)	0.435 (0.028)	1.00 (0.012)
relative variant transmissibility for waves 2+ ($v_{1}=1$)	$v_{2}$	1.11 (0.04)	0.90 (0.03)	1.02 (0.01)	0.98 (0.06)	1.30 (0.02)
	$v_{3}$	1.35 (0.06)	0.98 (0.06)	1.11 (0.03)	0.52 (0.02)	0.40 (0.01)
	$v_{4}$	1.77 (0.10)	1.33 (0.10)	1.41 (0.02)	0.75 (0.03)	0.65 (0.01)
activity contact rates (β)	$\beta^{Home}$	–	−10.0 (−)	−10.0 (−)	0.15 (1.18)	−10.0 (−)
	$\beta^{Trend}$	–	0.27 (0.06)	1.44 (0.08)	0.20 (0.08)	0.02 (0.002)
	$\beta^{Entert.}$	–	−0.02 (0.31)	5.73 (0.57)	0.46 (0.46)	−0.37 (−)
	$\beta^{Work}$	–	0.06 (0.07)	2.88 (0.32)	0.11 (0.13)	−0.14 (−)
	$\beta^{Transit}$	–	−0.22 (0.56)	3.92 (0.18)	0.02 (0.71)	1.29 (−)
	$\beta^{Grocery}$	–	4.80 (4.00)	0.71 (0.25)	−5.83 (5.37)	−0.01 (0.03)
	$\beta_{0}$	–	–	–	1.64 (0.72)	1.15 (0.02)
Variance parameter (u)		8821.30 (711.62)	5885.91 (469.99)	3947.20 (300.18)	2178.65 (162.68)	2189.57 (161.52)
−l(θ)		4124.7	4083.3	4010.7	3894.5	3895.6
# of parameters		8	14	14	15	15
AIC		8265.4	8194.6	8049.4	7819.0	7821.2

Notice from the likelihood and AIC values in Table 2 that including the GMR data leads to a significant improvement in fit. In particular, the trend component is highly significant across the different model setups. Still, the quality of fits is not entirely satisfactory, as waves 1 and 3 are only partially fitted, and the peak of wave 4 is underestimated. From our previous work on this data, we have obtained superior fits by introducing different reduction parameters to fit different levels of public health restrictions over the time periods they were known to be in effect. Ideally, the severity of public health restrictions on mobility should be reflected in a reduction in GMR aggregate indices over the duration of those restrictions. We can test the sensitivity of GMR as a proxy of epidemiologically-relevant mobility by allowing for a single, parallel drop in mobility index ${\tilde{M}}_{t}$ during the times of public health restrictions (2020-Mar-22 to 2021-Jun-14). We model this drop by parameter $\beta_{0}$ such that ${\tilde{M}}_{t}={\tilde{l}}_{lower}+{\tilde{l}}_{upper}f\left(h_{t}∙\beta +\beta^{H}-\beta_{0}I_{\left\{t\in \text{restrictions}\right\}}\right)$. We adjust the start and end dates of restriction measures to reflect a delay of ${incubation}_{t}+case\_delay$, which is the time it would take to begin seeing an effect in infection counts. Thus, $\beta_{0}$ estimates any extra effects from public health orders not captured by the Google data. These are likely changes in behavior other than isolation (e.g., social distancing in public places).

We fit the model including $\beta_{0}$ using the same two reduction scenarios. Fig. 2 shows a much improved fit to observed case data with the additional $\beta_{0}$ shift. This is confirmed quantitatively by the AIC values. For better illustration we have plotted the implied values of restriction level $K_{t}$ in the downscaled scenario, which can be interpreted as the fraction of current mobility compared to pre-pandemic times (Fig. A1 in the Appendix). Notice that even though the GMR data generally shows lower activity during public health restrictions, it fails to capture the full extent of the reduction in epidemiological contacts, as implied by the highly significant effect of $\beta_{0}$. The change in level $K_{t}$ under the tapered scenario is qualitatively similar (results not shown). One explanation of why the mobility metric may not be epidemiologically sensitive enough might be that categories defined by Google are too broad, and are not stratified by infection potential. For instance, the Retail and Recreation category includes bars and restaurants, where individuals stay in close contact for extended periods and engage in casual conversation, as well as large retail stores (e.g., Walmart), which typically do not offer nearly the same opportunity to transmit infection. Public health orders specifically targeted venues in the former category with closures, while allowing those in the latter to stay open (for good reason). It is entirely conceivable that individuals changed their behavior in response to such restrictions by going shopping more instead of going to restaurants. This change is likely to significantly impact case numbers, however may not have much effect on the GMR, which counts visits to both types of venues the same.

### 3.2 Categories comparison

Next, we wish to better understand how changes in activity levels for various categories affect epidemic spread. Firstly, based on our model formulation, the components of β are interpretable as the effect of an extra hour spent in that category on the average number of connections formed on that day. However, care should be taken in interpretation due to the non-linearity of ${\tilde{M}}_{t}$, as adding time increments to a category will not result in equal increments in contacts formed. This means that such inferences are only accurate around the best fit point in the parameter space. Looking at the estimates in Table 2, there is no consistency between the different model fits, and, in every case but one, the estimates are not significant, most likely due to multicollinearity among the GMR time series. For the best fitting model in Table 2, a test of $H_{0}:\beta^{E}=\beta^{W}=\beta^{T}=\beta^{G}$ versus $H_{A}\text{:}$ not all β are equal, is not significant, meaning that, at least under scenario 1, the trend term captures the full effect of the mobility data (recall that $h_{t}^{trend}$ represents the total time spent in any of the activity categories mentioned). For the fit under scenario 2 without $\beta_{0}$, surprisingly, all activities show statistically significant parameters. These results are appealing because they are in line with what we may expect intuitively: for instance, more contacts are formed spending time in entertainment and retail versus work; as well, spending time in transit results in more contacts than doing grocery shopping. These results to some degree corroborate the findings of Li et al. (2021), who conclude that increased visits to retail and recreation, and workplaces increase $R_{t}$ substantially more than visits to grocery and pharmacy, while spending more time at home or in parks decreases $R_{t}$. Arguably, the latter may simply be a result of having less time to spend in the contact-generating activities. They also find that increased volumes in transit stations result in even higher increases in $R_{t}$, but only in major cities, which is in line with our results.

Pursuing this line of inquiry further, we can estimate the impact of each category on the total epidemic burden. To assess this, we increase each component category of $h_{t}$ by 0.5 (or 30 minutes) at all time steps, and compute the extra number of infections compared to the original fit. From Table 3 we see that the most time-efficient activities in terms of generating infections are retail and recreation (a.k.a. entertainment), followed by transit stations and work. It is important to use caution, however, when interpreting these results, as the same parameters are not supported by the better fitting model including $\beta_{0}$. Thus, the significant relationships observed above may be due to overfitting, and hence the results in Table 3 should be viewed as exploratory.

**Table 3:** Sensitivity analysis of total infections generated by an extra 30 min spent in each category. Original fit is under the tapered scenario in Table 2, which has 10,168,410 infections.

Parallel upward shift in	Total infections	Δ infections	% change
Trend	10,758,758	590,348	5.8
Entertainment	12,781,442	2,613,032	25.7
Work	11,419,810	1,251,400	12.3
Transit	11,997,724	1,829,314	18.0
Grocery	10,453,118	284,708	2.8

### 3.3 Comparison between reduction scenarios

**Fig. 3:**
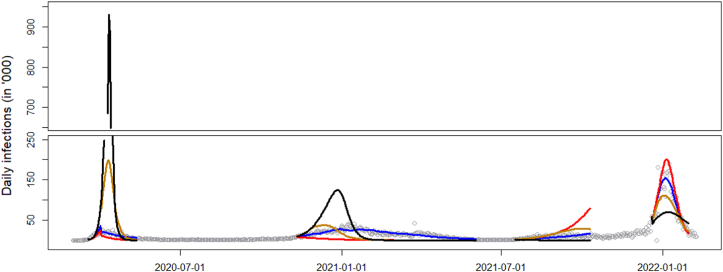
Daily projected number of infections under the three scenarios, matched for daily contact volumes: downscaled (blue), tapered (gold), top off (red). For reference, the infection curve if no precautions were taken (i.e., no change in mobility from baseline) is given in black.

An interesting observation from the best fitting models under scenarios 1 and 2 is that we get essentially the same quality of fit from very different model settings. One could easily (and incorrectly) conclude that there is no difference between the reduction scenarios. In order to compare the effectiveness of the different reduction scenarios for curve c(∙) at stopping epidemic spread, we need to keep both the epidemic model, as well as the mobility level ${\tilde{M}}_{t}$, fixed, and project what the infection curve would look like, if we only changed the reduction mechanism. In other words, we use the same overall daily loss of contacts regardless of scenario, and solve for the corresponding levels $K_{t}$ under each scenario separately. Then we compute and compare the curves starting from the same epidemiological parameters, but using the $K_{t}$ time series that was calculated under each of the three scenarios. The fixed parameter values are based on the scaled down scenario including $\beta_{0}$ (this had the best fit in Table 2), with the difference that we exclude the four category β’s, as they are not significant. The projected curves for each scenario are shown in Fig. 3, along with a curve for no reduction. As can be seen, all scenarios manage to curb the pandemic, which would otherwise peak at an extreme level during the first wave. However, the epidemic trajectories are quite different for each. To get a sense of their stopping power, in Table 4 we cumulate infections across all waves. We do the same starting from the tapered fit, as it is qualitatively similar, but implies a very different epidemiological model (in particular, a large fitted ν results in more reinfections and overall higher infected counts). In both assumed models, scenario 3 results in the highest reduction in cases. This is not surprising, as targeting the most connected individuals removes the most edges from the underlying network.

**Table 4:** Projected infections under each reduction scenario, starting from a common epidemiological model and assuming the same overall mobility volumes.

Assumed model	Infections without change in mobility	Infections assuming mobility effects in scenario 1	Infections assuming mobility effects in scenario 2	Infections assuming mobility effects in scenario 3
**Scaled down fit**	15,841,339	10,003,994	10,828,367	8,754,721
**Tapered fit**	25,656,809	18,503,509	10,097,984	5,861,201

## 4 Discussion

This paper presented a framework for integrating aggregate cell phone mobility data into a model of infectious disease spread. The most important contribution to the field is identifying the relationship between total contacts, or edges, in the population network, and the contact rate in the epidemiologic transmission model. Estimating the former from aggregate cell phone volume in the population allows us to infer how the latter fluctuates in response to changing public behavior and policies. The paper also demonstrated how different scenarios for reduction in average number of connections could have a profound impact on reducing the total burden of disease. The potential of this research is quite enticing; for instance, it allows public health practitioners to answer questions such as “if we were to mandate working from home for two days a week, what would the impact be on spread?” With a well-calibrated model, this can be predicted by simply reducing $H_{t}^{W}$ by 40% and predicting the new epidemic trajectory with all other variables kept at current levels. The ability to estimate how the contact rate curve changes in response to policy can be invaluable in designing future interventions.

The availability of cell phone data, which can inform aggregate network statistics, is welcome in infectious disease modeling. However, there is room for growth in both the quality of this data and in how it is modeled. Starting with the data itself, the Google Community Mobility Reports were introduced as another one of the Google suite of products with the general aim of providing “insights into changes in mobility patterns” (Aktay et al., 2020). There is no indication that this was intended to be used by epidemiologists, so we can assume that infectious disease modeling was not the intended application. However, a mobility metric based on cell phone data is certainly a much needed tool for modellers in epidemiology, as this paper and others (e.g., Chang et al., 2021) have shown. A couple of recommendations for any future iterations of GMR to make them more useful as research tools are as follows: 1) keep a single baseline, as opposed to changing the baseline according to the day of the week. Not having an explicit baseline for each day introduces unnecessary variability in the dataset (in the present paper, we made the assumption that all weekdays are alike, as are Saturday and Sunday). 2) Refine the categories to be more relevant to the spread of infection; in particular, points of interest that were specifically targeted by public health orders, such as dining and entertainment venues, should be reported separately from other retail and recreation categories. 3) An important limitation of this data is that it does not give absolute magnitudes of cell phone traffic, but only relative volumes. This is a barrier that prevents the complete identification of the network effect from the pathogen transmissibility effect.

**Fig. 4:**
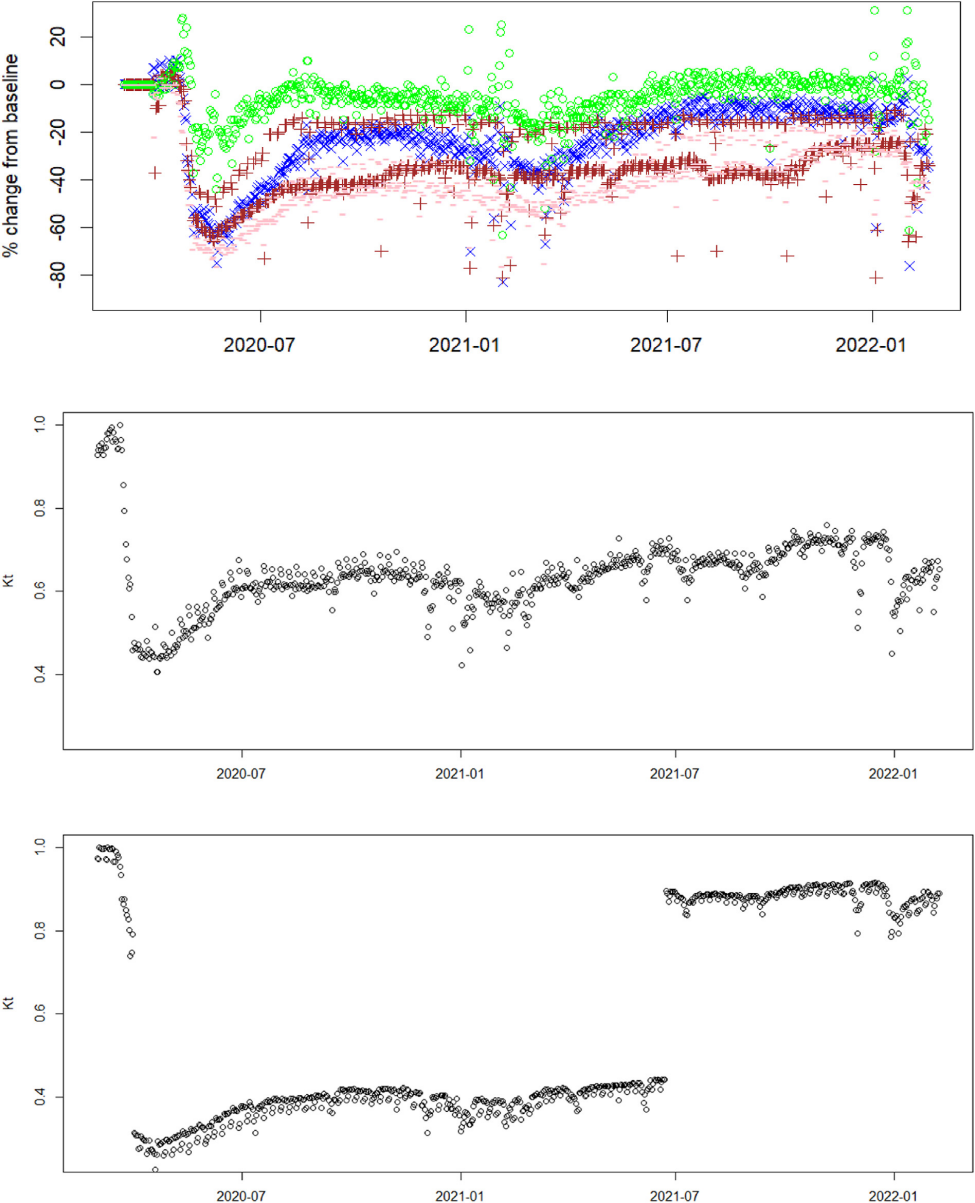
(top) Percentage daily changes in mobility from Google for categories: retail and recreation (x), workplaces (+), grocery & pharmacy (o), and transit stations (−). Restriction level $K_{t}$ implied by the fitted model under the downscaled scenario: (middle) excluding $\beta_{0}$ in the fit; (lower) including $\beta_{0}$.

In addition to improvements in quality of the input data, future efforts will need to focus on how to model and interpret mobility data. Unless working with very granular spatial data, which requires large, intractable models, aggregate mobility data will necessarily collapse different behavioural categories of individuals into single metrics. As a result, having insight into such patterns at the individual and population levels will be necessary to make the best use of this data. For example, there are two noticeable patterns in the implied restriction level $K_{t}$ in Fig. 4: 1) a general drop compared to pre-pandemic levels of more than 20%, from which the population does not recover; and 2) three lows corresponding roughly to the three largest COVID-19 waves. There are a couple of insights here: first, that there is likely a risk-averse segment of the population whose activity does not bounce back, even after official restrictions have been lifted. Second, given that the data strongly favors a large reduction in $K_{t}$ during the mandated orders, as shown by our fits including $\beta_{0}$, we would speculate that these three visible dips had a greater impact than can be captured on a linear or logistic scale. Perhaps two behaviorally different segments of the population – one segment that takes drastic measures to isolate even when the risk is low, and another one that responds only when the risk is high, or when forced to –, should be modeled separately. In any case, more research is needed to understand how individual compliance or risk aversion can be used to inform infectious disease models.

## Declaration of competing interest

The authors declare that they have no known competing financial interests or personal relationships that could have appeared to influence the work reported in this paper.

## Figures and Tables

**Table A1:** Average hours per day spent in activities related to the GMR categories, from the American Time Use Survey (ATUS). Figures are for the year 2019 and refer to the civilian noninstitutional population of ages 15 and over. Note that numbers for 2020 were not produced.

Activity category	Average hours per day, civilian population	
	Weekdays	Weekends and holidays
Retail & Recreation	0.98	1.86
Grocery	0.09	0.13
Work	4.63	1.26
Travel (overall)	1.2	1.11

**Table A2:** Algorithm to generate an epidemic curve from a given parameter set.

Steps	Compute an epidemic curve starting from $\theta =\left(a_{1},a_{2},a_{3},\nu ,v_{2},v_{3},\ldots ,\beta \right)$
1.	Set ${\tilde{l}}_{lower}=\tilde{c}\left(0\right)$ for scenarios 2 & 3, and ${\tilde{l}}_{lower}=0$ for scenario 1. Set ${\tilde{l}}_{upper}=\left[\int_{0}^{1}\tilde{c}\left(s\right)ds-{\tilde{l}}_{lower}\right]/f\left(\max_{t}\left(h_{t}∙\beta +\beta^{H}\right)\right)$
2.	Compute the mobility metric ${\tilde{M}}_{t}={\tilde{l}}_{lower}+{\tilde{l}}_{upper}f\left(h_{t}∙\beta +\beta^{H}\right)$.
3.	Solve Equation (7) for $K_{t}^{*}$, for each t. E.g., in scenario 1, $K_{t}^{*}={\tilde{M}}_{t}/\int_{0}^{1}\tilde{c}\left(s\right)ds$.
4.	LOOP FOR t=1..T
5a.	If this is the first wave, compute $R_{t}$ as in the SIR setting, under the chosen scenario. E.g., in scenario 2, $R_{t}=S_{t}\times \tilde{c}\left(K_{t}^{*}S_{t}\right)$. OR
5b.	In subsequent wave i AND setting is SIRS, compute $S_{-1},S_{0}$ and then $R_{t}=v_{i}S_{t}{\tilde{c}}^{w}\left(S_{t}/S_{0}\right)$. Note that $\tilde{c}$ in formula (4) depends on scenario (as in 5a).
6.	Compute $y_{t+1}=R_{t}\sum_{s=1}^{M}w_{s}y_{t+1-s}$.
7.	Update the susceptible fraction: $S_{t+1}=S_{t}-\frac{y_{t+1}}{\rho_{t}N}$.
8	End LOOP

**Table A3:** Algorithm to predict a hypothetical scenario different from the one being fit.

Steps	Fit the model under scenario X, and predict under scenario Y
1.	Optimize the epidemic curve under scenario X over the parameter space $\theta =\left(a_{1},a_{2},a_{3},\nu ,v_{2},v_{3},\ldots ,\beta \right)$ to obtain the MLE $\hat{\theta_{X}}$. The optimization is subject to the following restrictions, to ensure a more homogeneous fit between scenarios: − If X = Scenario 1, set ${\tilde{l}}_{lower}=\tilde{c}\left(0\right)$ in step 1 of Algorithm A1. − Set $\beta^{E}=\beta^{W}=\beta^{T}=\beta^{G}=0$ if not significant, to reduce the chance for multicollinearity.
2.	Compute the epidemic curve (as in A2) for parameter value $\hat{\theta_{X}}$, by only changing the reduction mechanism to scenario Y.
3.	Convert between projected infections and projected cases, as needed, using the formula ${cases}_{t}=\rho_{t}\times {infections}_{t}$.

## Appendix

*Solving Equation*(7)*in the tapered case*. To prove that $\int_{0}^{1}\tilde{c}\left(s;\;K_{t}\right)\text{ds}=\frac{\int_{0}^{K_{t}}\tilde{c}\left(s\right)\text{ds}}{K_{t}}$, start from $\tilde{c}\left(s;\;K_{t}\right)=\tilde{c}\left(K_{t}s\right)$. Then, using basic calculus:

$$\begin{array}{rr} \int_{0}^{1}\tilde{c}\left(s;\;K_{t}\right)ds & =\int_{0}^{1}\;\tilde{c}\left(K_{t}s\right)ds=\\ & =\int \frac{\tilde{c}\left(y\right)}{K_{t}}dy={\frac{1}{K_{t}}\int \tilde{c}|}_{y/K_{t}=0}^{y/K_{t}=1}\\ & =\frac{1}{K_{t}}\left[\int \tilde{c}\left(K_{t}\right)-\int \tilde{c}\left(0\right)\right]\\ & =\frac{\int_{0}^{K_{t}}\tilde{c}\left(s\right)ds}{K_{t}}\text{,} \end{array}$$

where $\int \tilde{c}\left(x\right)$ is the antiderivative of $\tilde{c}$, evaluated at x.

Note further that for $\tilde{c}\left(S_{t}\right)=a_{1}e^{-a_{2}{\left(1-S_{t}\right)}^{a_{3}}}$ as used in the text, we have $\int_{0}^{x}\tilde{c}\left(s\right)ds=\frac{a_{1}\left\{-\gamma \left(\frac{1}{a_{3}},a_{2}{\left(1-x\right)}^{a_{3}}\right)+\gamma \left(\frac{1}{a_{3}},a_{2}\right)\right\}}{a_{3}{a_{2}}^{1/a_{3}}}$, where γ(x,a) is the lower incomplete gamma function.

*Model implementation details.* The model was implemented in R v.4.3.2, and the code is adapted from the source code found in R package *REffectivePred* (Romanescu, 2024b). We set ${\tilde{l}}_{lower}=\tilde{c}\left(0\right)$, which is necessary to solve for $K_{t}$ in scenarios 2 and 3, and ${\tilde{l}}_{lower}=0$ in scenario 1. For all three scenarios, we set ${\tilde{l}}_{upper}=\left[\int_{0}^{1}\tilde{c}\left(s\right)ds-{\tilde{l}}_{lower}\right]/n_{c}$, where $n_{c}=\max_{t}f\left(h_{t}∙\beta +\beta^{H}\right)$ is a normalizing constant. This guarantees a consistent range between the mobility metric and the contact rate curve, ensuring that Equations (8), (9), (10) have solutions for each day t. As well, this particular choice implies that the maximum of ${\tilde{M}}_{t}$ corresponds to no restrictions ($K_{t}=1$); this is meaningful for the dataset considered, which covers a time period before the pandemic. If fitting a time period that falls entirely under restrictions, then $n_{c}$ could be set to 1.

Equations (9), (10) are solved numerically for $K_{t}$ using *uniroot* solver in R. The lower incomplete gamma function used in the integral for functions $\tilde{c}$ is computed via R function *gammainc*. Optimization of model parameters is done by maximizing the log-likelihood via *optim* function in R, using gradient descent. The likelihood function is based on the model for infections $y_{t}/\rho_{t}∼N\left(f_{t}\left(\theta \right)/\rho_{t},uf_{t}\left(\theta \right)/\rho_{t}\right)$, where $f_{t}$ denotes the epidemic curve computed by the model on day t. The fits shown in the main text correspond to the MLE $\hat{\theta }$. Due to the relatively large number of parameters, optimization might present different areas of the parameter space with comparably good fit. As a general strategy, we start the optimizer from the best fits reported for this dataset in (Romanescu et al., 2023) and may place interval restrictions on parameters common with that model in order to preserve those fits qualitatively. In this way, the optimizer searches for the best β parameters (new to this model) that are consistent with those fits. As a disclaimer, due to the large parameter space and inherent identifiability problems with this class of models, it may be possible that results shown represent local as opposed to global maxima of the likelihood function.
